# Spillover of a hepatitis A outbreak among men who have sex with men (MSM) to the general population, the Netherlands, 2017

**DOI:** 10.2807/1560-7917.ES.2018.23.23.1800265

**Published:** 2018-06-07

**Authors:** Ingrid HM Friesema, Gerard JB Sonder, Mariska WF Petrignani, Annemarie E Meiberg, Gini GC van Rijckevorsel, Wilhelmina LM Ruijs, Harry Vennema

**Affiliations:** 1 Centre for Infectious Diseases, Epidemiology and Surveillance, Centre for Infectious Disease Control, National Institute for Public Health and the Environment (RIVM), Bilthoven, the Netherlands; 2 Department of Infectious Disease Control, Public Health Service Amsterdam (GGD), Amsterdam, the Netherlands; 3 National Coordination Centre for Communicable Disease Control, Centre for Infectious Disease Control, National Institute for Public Health and the Environment (RIVM), Bilthoven, the Netherlands; 4 Centre for Infectious Diseases Research, Diagnostics and Screening, Centre for Infectious Disease Control, National Institute for Public Health and the Environment (RIVM), Bilthoven, the Netherlands

**Keywords:** hepatitis A, outbreaks, surveillance, MSM, spillover

## Abstract

Since 2015, outbreaks of hepatitis A among men who have sex with men (MSM) have been reported worldwide. To examine the impact of these MSM outbreaks in the Netherlands, we combined notification and epidemiological data with sequence analysis. Our results show the hazards of outbreaks within risk-groups spilling over into the largely susceptible general population. One third of the outbreak-related hepatitis A virus genotypes were detected in non-MSM cases.

Hepatitis A outbreaks among MSM are ongoing in Europe and the rest of the world [1-10]. Genetic sequencing of these outbreaks revealed three independent strains (VRD_521_2016 (UK/Spain), RIVM-HAV16–090 (EuroPride) and V16_25801 (Germany)) circulating within the outbreaks, all of which were genotype IA [1,2,4,5,9]. Between September and December 2016, the first eight Dutch MSM outbreak–related cases were reported [4]. In the Netherlands, sequence data for more than 70% of the hepatitis A cases were available enabling us to assess the magnitude of the outbreak and the spillover to the general population.

## Notification and sequence data

Hepatitis A is a notifiable disease in the Netherlands. Laboratories and physicians report hepatitis A virus (HAV) infections to the regional Public Health Service (PHS). After notification, the PHS gathers information on basic demographics, disease onset, symptoms and hospitalisation, most likely source of infection (MSM contacts, related cases, travel history) and, if there is no other obvious source of infection, the consumption of specific food items is also collected. For national surveillance, PHS report these data anonymously to the Netherlands National Institute for Public Health and the Environment (RIVM).

All laboratories are requested to send serum and/or stool samples to the RIVM for molecular analysis. HAV-positive samples are analysed by sequence analysis of a 460 nt PCR fragment in the VP1/P2A region, according to a shared protocol available through the hepatitis A Laboratory-Network (HAVNET) [11].

Unless specified otherwise, we labelled all three MSM-related strains as ‘MSM-strains’. Clusters of cases were identified using information about related cases in the notification form. When sequence data of at least one case within a cluster was available, we assumed that all cases within that cluster were infected by the same strain. This assumption was supported by analysis of clusters with at least two cases with sequence data.

## Hepatitis A cases in 2017

In 2017, 374 hepatitis A cases were notified to RIVM including 293 men and 81 women with an age range of 1 to 78 years. A total of 122 cases were reported as part of a cluster (50 clusters). Of the men, 174/293 (59%) reported to be MSM and 109 non-MSM; for 10 men, the MSM status remained unknown. Sequence data was available for 317 cases (84.8%) and 18 cases within a cluster lacked sequence data but were assigned to a strain based on sequence data from other cases within that particular cluster. This left 39 cases without strain information (10.4%).

**Figure 1:**
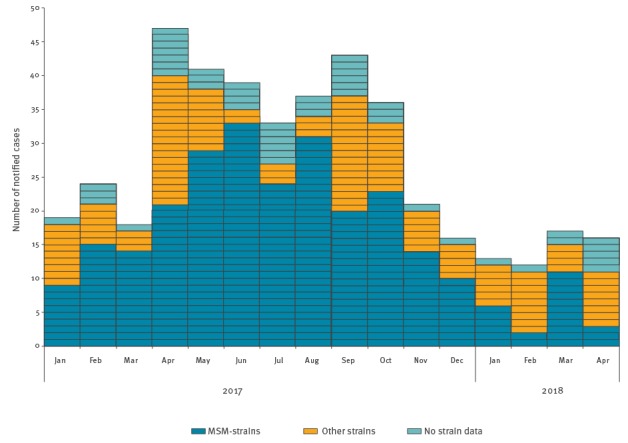
Number of notified cases (n=432) stratified to MSM-strains (n=265), other strains (n=119) and no strain data (n=48) per month of disease onset, January 2017–April 2018

In total, 243 cases were infected with an MSM-strain and 92 were infected with another HAV-strain. The distribution of the cases per month, segmented into type of strain found, is shown in Figure 1. Most cases fell ill between April and October, with peaks of non-MSM HAV-strains in April and September. In Europe, the MSM outbreak peaked between March and July, and there was a decrease hereafter although the decrease in the last months could be due to reporting delays [12].

## Cases with ‘MSM-strains’

Since October 2014, no cases of hepatitis A among MSM were reported in the Netherlands. During the second half of 2016, there were nine MSM cases reported: one was infected with a ‘non-MSM strain’ after travelling, seven were infected with an MSM-strain and for one, who was epidemiologically likely related to the MSM outbreak, there was no strain information available [4]. All 158 MSM with sequence data in 2017 were infected with an MSM-strain, compared with 57% of the non-MSM (55/96) and 32% of the women (23/73). Seven of eight men of whom the MSM status was unknown were also infected with an MSM-strain. Within the MSM-strains, the distribution was VRD_521_2016 (n = 130; 54%), RIVM-HAV16–090 (n = 92; 38%) and V16_25801 (n = 21; 9%). Of the 92 other HAV strains, most were genotype IA (n = 49) or genotype IB (n = 35), and eight strains were typed as IIIA.

**Figure 2:**
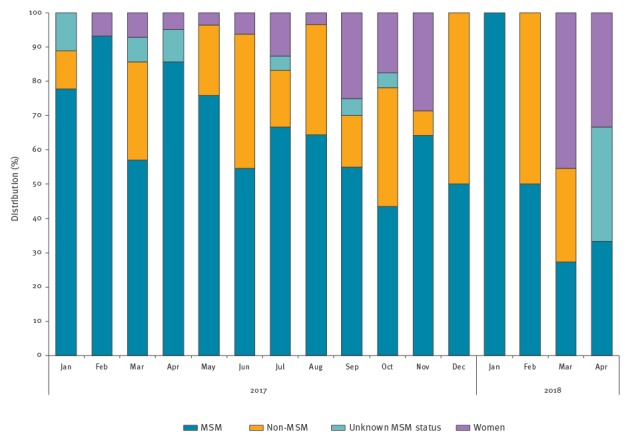
Distribution of the MSM-strains (n=265) in MSM (n=169), non-MSM (n=59), unknown MSM status (n=8) and women (n=29) per month, January 2017–April 2018

The distribution of MSM-strains in MSM, non-MSM, unknown MSM status and females between January 2017 and April 2018 is given by month in Figure 2. The first MSM-strains in non-MSM cases were seen in January 2017, but proportions increased as from June.

## Comparison of outbreak-related and other cases

In the Table, characteristics of cases with MSM-strains and with other strains are compared. Statistical significance was assessed using chi-squared test (p < 0.05).

The median age for cases with MSM-strains was 35 years (range 2 to 78 years) versus 23 years (range 1 to 70 years) for cases with non-MSM strains. Nine children were infected with an MSM-strain, while the majority of children (n=32) were infected by a non-MSM strain. More than one third (36%) of the infections with the non-MSM strains were contracted in countries with high prevalence of hepatitis A. Both MSM and non-MSM cases contracted an MSM-strain less often when travelling abroad than cases infected with other strains; almost none of the cases infected with an MSM-strain (2%) had travelled to an endemic country. Thus, these cases mainly contracted the disease in the Netherlands or within other non-endemic countries. This is consistent with findings from other European countries [1,8,9]. MSM cases were more often born outside the Netherlands than other hepatitis A cases. A similar picture was reported by Rodríguez-Tajes et al. [8] in hospitalised cases in Barcelona, where cases were more often born outside Spain. No clear explanation was found.

**Table:** Characteristics of cases separated to strains and MSM status, the Netherlands, 2017

Characteristics	MSM-strains								Other strains					
	Men				Women (n=23)		Total (n=236)		Men		Women (n=50)		Total (n=91)	
	MSM (n=158)		Non-MSM (n=55)						Non-MSM (n=41)					
	**median**	**range**	**median**	**range**	**median**	**range**	**median**	**range**	**median**	**range**	**median**	**range**	**median**	**range**
Age (years)	34.5	19–78	37	2–70	37	6–69	35	2–78	23	3–70	23.5	1–70	**23^a^**	**1**–**70^a^**
	**n**	**%**	**n**	**%**	**n**	**%**	**n**	**%**	**n**	**%**	**n**	**%**	**n**	**%**
Children	0	0	5	9	4	17	9	4	11	27	21	42	**32^a^**	**35^a^**
<5 years	0	0	2	4	0	0	2	1	1	2	2	4	3	3
5–9 years	0	0	1	2	3	13	4	2	3	7	11	22	14	15
1017 years	0	0	2	4	1	4	3	1	7	17	8	16	15	16
Hospitalisation	35	22	15	27	8	35	58	25	7	17	12	24	19	21
Born in the Netherlands	92	58	40	72	17	74	149	63	34	83	37	74	**71^b^**	**78^b^**
Travel history	34	22	11	20	7	30	52	22	14	34	21	42	**35^b^**	**38^b^**
Endemic country	3	2	1	2	1	4	5	2	14	34	19	38	**33^a^**	**36^a^**
Part of epi-cluster (number of clusters)	35 (19)	22	21 (13)	38	13 (10)	57	69 (32)	29	19 (8)	46	28 (12)	56	**47^a^**(12)	**52^a^**

^a^ p < 0.0001; ^b^ p < 0.01; Males with strain information but without known MSM status (n=10) were excluded from the table. There were no MSM men infected with other strains.

The most common route of transmission of HAV within the MSM outbreak is directly person-to-person via the faecal-oral route, but it can also be transmitted indirectly via surfaces and food [13]. Only a small proportion of the MSM cases (22%; 35/158) reported having contact with a known hepatitis A patient. This corresponds with the reported, often anonymous and once-only, sexual contacts among MSM, hampering finding secondary cases and distributing post-exposure prophylaxis given to close contacts. As at least six MSM cases reported not having had sexual contacts in the two months before illness, not all hepatitis A infections within the MSM scene are transmitted sexually. Nevertheless, these cases had been visiting gay venues, such as gay bars and clubs, pointing towards environmental contamination. Similar observations have been reported before [14,15].

## Discussion

A decreasing trend in HAV seroprevalence and incidence has been seen in Europe over the last few decades [16], creating increasing susceptible populations. The Netherlands is classified as a country with a high susceptibility profile to hepatitis A, due to low prevalence of HAV and low vaccination status [16]. It is not surprising, therefore, that an outbreak within a risk group, in this case MSM, spreads easily into the general population. Of the 243 cases with MSM-strains in 2017, 65% were MSM, so for every two MSM cases one person from the general population will also be infected. Spillover of the infection was more common to men than to women. This could potentially be explained by men not reporting to be MSM, but it could also be hypothesised that men have more casual contact with each other than with women; public toilets being gender-specific, for example. As children were rarely infected with an MSM-strain, it supports the belief that transmission mainly occurred between adults in adult settings.

The number of HAV cases in 2017 assigned to the MSM outbreak was 243 cases; 92 cases were unrelated to this outbreak. The amount of the non-outbreak cases is comparable to the amount of reported cases in 2011 to 2016, i.e. 80–124 cases yearly. Thirty-nine cases could not be assigned, as no strain information was available, but no bias towards sex, MSM status or region was seen.

The MSM outbreak is fading out, but is not over yet, as demonstrated by a regional cluster of 8 cases in the general population seen in March 2018 (source of the infection was not found). In the first four months of 2018, 22 cases were confirmed as having an MSM-strain. Adding these cases to the eight cases confirmed in 2016 and the 243 cases in 2017, results in a total of 273 MSM outbreak–related cases in the Netherlands between 1 June 2016 and 30 April 2018. In Europe, a total of 4,101 outbreak-related cases were counted between 1 June 2016 and 19 March 2018; overall, four times more HAV cases were reported in 2017 (20,089 cases) compared with 2012 to 2015 (average 5,648 cases) [12]. Only symptomatic laboratory-confirmed cases were included, resulting in an underestimation of the true extent of the outbreak. In addition, although the majority of older children and adults will show symptoms, with jaundice occurring in up to 70% of the cases [17], some cases will only have mild symptoms or be asymptomatic and could therefore be missed.

## Conclusion

The availability of both epidemiological and sequence data for the majority of the cases (89.6%) made it possible to investigate the spillover of the infection from MSM to the general population and to examine differences between outbreak-related and other cases. This MSM outbreak highlights the hazards of the introduction of HAV in a population with increasing numbers of susceptible citizens, not only for high-risk groups, but also for the general population. In the current Dutch outbreak, about one third of the cases with an MSM-strain in 2017 did not have an MSM link. What was particulary interesting was finding that spillover to children was rare, whichcould potentially be explained by specific transmission networks within the outbreak.
